# Sport-Specific Risks of Osteochondritis Dissecans Across Athletic Disciplines: A Narrative Review

**DOI:** 10.3390/healthcare13151857

**Published:** 2025-07-30

**Authors:** Tomasz Poboży, Michał Derczyński, Wojciech Konarski

**Affiliations:** 1 Independent Researcher, 00-001 Warsaw, Poland; tomasz.pobozy@onet.pl (T.P.); michalderczynski@gmail.com (M.D.); 2 Medical Rehabilitation Center, Sobieskiego 47D, 05-120 Legionowo, Poland

**Keywords:** osteochondritis dissecans, OCD, football, basketball, baseball

## Abstract

Osteochondritis Dissecans (OCD) is a joint condition characterized by damage to the surface of the joint and the underlying subchondral bone, leading to early-onset osteoarthritis. It predominantly affects the knee, elbow, and ankle, with higher prevalence in juveniles actively participating in sports, which complicates the condition due to slow healing processes and prolonged restrictions on physical activities. This review aims to summarize current knowledge on OCD in athletes, with emphasis on sport-specific risk factors, diagnosis, and treatment, to support clinical decision-making and future research. We conducted searches in the PubMed and Embase databases, covering the period from 2014 to 2024. The keywords used in the search covered most common sports in combination with term osteochondritis dissecans. This review examines the impacts of various sports on the development of OCD, analyzing prevalence and risk factors, with a focus on sports-specific risks across athletic disciplines like football, basketball, baseball, and gymnastics. The significance of early detection, intervention, and sport-specific conditioning is underscored to prevent the condition and manage it effectively. Moreover, the review highlights the positive prognosis for athletes, particularly adolescents, recovering from OCD, with a high rate of return to sport. Understanding the sports-specific risks, ensuring early intervention, and adopting a cautious, stepwise return to sport are critical for managing OCD effectively, thereby safeguarding the health and careers of athletes.

## 1. Introduction

Osteochondritis dissecans (OCD) represents a challenging and potentially debilitating joint condition affecting individuals across various age groups, particularly active juveniles and athletes. The condition is characterized by the separation of a segment of cartilage and subchondral bone, resulting in local ischemia and lead to necrosis of the affected area [1,2,3].

A multicenter prospective cohort study found that OCD lesions most commonly occur in the knee, elbow, and ankle, with lesions more prevalent among juveniles than in adults [4,5]. The prevalence of knee OCD is 2.3–31.6 per 100,000, while the elbow and ankle have a prevalence of 6.0 and 4.6 per 100,000, respectively [4,6,7,8]. Most patients with OCD (55–60%) actively participate in sports. Young athletes often find the condition frustrating because of its slow healing process, prolonged restrictions on sports activities, and ongoing pain or discomfort [6,9].

The exact etiology of OCD remains a subject of debate, but it is widely believed to involve a combination of developmental and environmental factors, including repetitive trauma or stress to the joint, poor blood supply to the area, and possibly genetic predisposition. The continual stress can cause vascular problems and provoke a response in the subchondral bone, potentially interfering with the healing process of the bone’s trabeculae and impairing the bone’s ability to regeneration [10,11].

The importance of early diagnosis and appropriate management of OCD cannot be overstated, as delayed or inadequate treatment can lead to joint deterioration, early onset osteoarthritis, and long-term functional impairment.

The objectives of this narrative review include providing an overview of the current understanding of the epidemiology, etiology, and management strategies for OCD, with a particular focus on the risks associated with selected athletic disciplines. We have focused on baseball, football, basketball, and gymnastics because there are the most reports in the context of these sports [12,13,14,15,16,17]. By enhancing awareness and understanding of OCD in the context of sports, we aim to facilitate timely diagnosis, improve treatment outcomes, and ultimately help athletes return to their activities. There are many individual reports about OCD in various sports, but there is a lack of a consolidated source of knowledge [12,13,14,15,16,17].

Therefore, the aim of this review is to synthesize existing knowledge on OCD in athletes by highlighting sport-specific risk factors, diagnostic approaches, and treatment strategies, thereby serving as a practical and comprehensive reference for clinicians, researchers, and sports professionals involved in the care of affected individuals

## 2. Methods

We conducted searches in the PubMed and Embase databases. The keywords used in the search covered most common sports in combination with term osteochondritis dissecans. We searched keywords in titles and abstracts. The search phrase used was as follows: (baseball[Title/Abstract] OR basketball[Title/Abstract] OR gymnastics[Title/Abstract] OR football[Title/Abstract] OR soccer[Title/Abstract]) AND (“osteochondritis dissecans”[Title/Abstract]) In addition to searching the database, we examined the bibliographies of the relevant articles.

**Inclusion criteria:**

- Original articles published in English between 10 November 2014 and 2024;
- Studies focused on osteochondritis dissecans related to baseball, football, basketball, or gymnastics;
- Study types: case reports, case series, retrospective and prospective cohort studies, and systematic reviews based on original data, and meta-analysis.

**Exclusion criteria:**

- Studies not focused on the specified sports;
- Non-English publications;
- Editorials, letters, expert opinions, reviews not based on original data, and conference abstracts without full-text availability.

## 3. Results

### 3.1. Baseball Is Described as the Sport with a Significant Contribution to OCD

The prevalence of OCD among baseball players is estimated to be between 1.2% and 7.4% [12,18,19,20]. OCD is a result of valgus overload, which increases contact pressure in the radiocapitellar joint. The cause of OCD in the humeral capitellum might be due to repetitive compression forces on the humeroradial joint, leading to microtrauma that can result in changes to the articular cartilage and stress reactions in the subchondral bone of the humeral capitellum [20,21]. In their laboratory study on cadaver specimens, Rotman et al. [22] described a novel phenomenon: the motion of the radial head across the capitellum during rapid extension, typical in actions like baseball pitching, lags compared to that observed during passive elbow movement. Understanding this mechanism is crucial as the first step toward preventing OCD.

Details of studies involving baseball players are presented in Table 1. Commonly reported risk factors of OCD include repetitive valgus stress and compression, limited vascular supply to the lateral capitellum, young age with open physes, early sports specialization, and high training volume. Clinically, extension pain and limited range of motion are frequent findings. In the youth baseball, the role of the pitcher is frequently assumed by those players who possess a rich background in the sport and can deliver pitches at high velocities. This group, as a result, is subject to substantial chronic elbow stress. It has been postulated that pitchers, due to the significant physical demands placed on their upper limbs during play, suffer from an increased rate of elbow pain in comparison to their counterparts in other positions.

A large-scale Japanese study [23] conducted between 2006 and 2020 investigated the prevalence of capitellar OCD in elementary school baseball players from Tokushima, using a standardized screening protocol. Over 14,000 players were screened with ultrasonography between 2011 and 2020, identifying 195 cases of capitellar OCD, corresponding to a prevalence of 1.4%. Most lesions were asymptomatic, with only 10.8% of affected players reporting lateral elbow pain. Radiographically, 73.3% of lesions were stage I, 24.1% stage II, and 2.6% stage III. No cases were diagnosed before the fourth grade, and the prevalence increased with age. Ultrasonographic screening was significantly more effective than physical examination alone (confirmation rate: 65.8% vs. 1.9%). The findings support the utility of early ultrasound-based screening to facilitate early detection and conservative management.

Teruya et al. [19] discovered that a restricted range of elbow extension and pain during elbow extension could be linked to the development of OCD. These insights not only corroborate previous medical understanding but also set the stage for targeted preventative strategies.

In the study by Clark et al. [24], lesions in baseball players had a mean sagittal inclination angle of 55.1° ± 11.9°, similar to non-upper extremity athletes, but significantly less lateral containment (2.5 ± 1.6 mm, *p* < 0.001). This suggests that the valgus forces in baseball players result in lesions that are more laterally positioned and less contained, highlighting distinct pathomechanical influences compared to both gymnasts and non-upper extremity athletes.

In the comprehensive cross-sectional and case–control study by Kida et al. [12], which included 2433 baseball players (average age, 14.5 ± 1.5 years), OCD of the humeral capitellum was identified in 82 (3.4%) of the elbows using ultrasonography. The analysis revealed that players with an OCD lesion started playing baseball at a younger age (*p* = 0.016), had engaged in competitive play for a longer duration (*p* = 0.0013), and reported more instances of both current (*p* = 0.0025) and previous (*p* < 0.0001) elbow pain compared to players without a lesion.

In young players, conservative treatments are frequently advised with good outcomes [25]. In the notable case report, Tajika et al. [26] reported successful nonoperative treatment of OCD of the humeral capitellum in a young baseball player (12 years old) with advanced skeletal maturity. They concluded that conservative treatment could be a viable option for young baseball players if lateral new bone formation within the OCD lesion is observed on radiographic images, even when the epiphyseal lines of the capitellum and lateral epicondyle are closed. A unique case report by Takata et al. [27] described identical capitellar OCD lesions in 11-year-old monozygotic twin baseball players, highlighting a potential genetic predisposition in the etiology of this condition. Despite initial conservative management, both athletes required autologous osteochondral graft transplantation following lesion progression. The near-identical location, morphology, and clinical course in genetically identical individuals strongly support the hypothesis that hereditary factors may contribute to susceptibility, alongside mechanical stress from repetitive throwing. Both players successfully returned to competitive baseball within four months postoperatively, with no recurrence of symptoms.

In more complex cases or in older patients, surgical methods such as arthroscopy, microfracture, osteochondral autograft transplantation, or fragment fixation method using absorbable pins may be employed. Studies report excellent return-to-play rates after surgical treatment over long-term follow-ups [28,29,30].

**Table 1:** OCD in baseball players: summary of findings.

Study	Population	Diagnostic Method	Location	Risk Factors	Treatment and Outcome
Teruya et al., 2023 [23] Prospective screening	135 elementary school students screened 10 diagnosed (7.4%)	Physical exam and ultrasound	Humeral capitellum	Extension pain Limited ROM in extension	ND
Clark et al., 2024 [24] Cross-sectional study	30 pediatric and adolescent patients (31 elbows) Mean age: 13.5 years 100% male	Radiographs, MRI	Humeral capitellum	Repetitive valgus shear forces Limited vascular supply to the lateral capitellum Adolescent age group, still in skeletal growth	ND
Kida et al., 2014 [12] Cross-sectional and case–control study	A total of 2433 baseball players (mean age, 14.5 ± 1.5 years). Diagnosed 82 (3.4%) elbows	Ultrasonography, radiographs	Humeral capitellum	Playing baseball at an earlier age Longer duration of competitive play	ND
Matsuura et al., 2024 [23] Prospective screening	Total players screened (2006–2020): 23,153 OCD cases identified (2011–2020): 195	Ultrasonography, radiographs	Humeral capitellum	High training volume	Conservative treatment preferred for early-stage
Leal et al., 2024 [29] Systematic review	136 patients (138 elbows) 12.7–15.7 years	Radiographs, MRI	Humeral capitellum	Repetitive valgus stress on the elbow during throwing motions Participation in overhead sports during skeletal immaturity Subchondral bone stress and limited vascularity of the lateral capitellum Young age	arthroscopic microfracture with or without debridement Return to sport ranged from 60% to 100%, typically within 4 to 5 months
Tajika et al., 2021 [26] Case report	12-year-old male	Ultrasonography, radiographs	Humeral capitellum	Repetitive valgus stress and compression Genetic predisposition	Conservative management Radiographic and ultrasound-confirmed healing within 12 months. No surgical intervention required
Takata et al., 2021 [27] Case report	Two 11-year-old identical twins	Ultrasonography, MRI, radiographs, CT		Early sports specialization in baseball, especially pitching Repetitive valgus overload and compression of the radiocapitellar joint	Initial conservative treatment failed, and both twins underwent autologous osteochondral graft transplantation from the knee after lesion progression Rehabilitation included early ROM exercises and return to competitive baseball within 4 months, with no symptoms at 6-month follow-up
Rothermich et al., 2023 [28] Retrospective study	107 adolescent patients (mean age: 15.2 years) Treated arthroscopically	Clinical evaluation and imaging	Humeral capitellum	Repetitive valgus stress	Arthroscopic surgery only (no open procedures) 93% return-to-play rate
Sasanuma et al., 2020 [30] Cohort study	32 male baseball players, aged ≤15 years (mean age: 14.1)	ND	Humeral capitellum	ND	Osteochondral autograft transplantation 93.8% return to sport

CT—Computed Tomography; MRI—Magnetic Resonance Imaging; ND—Not Described.

### 3.2. Football

Football players may experience injuries from both contact and non-contact incidents, but the most frequent cause of pain is often overuse. The most commonly affected areas include the knees, ankles, heels, and feet, which are mechanically vulnerable points. In adults, overuse injuries typically affect tendons, muscles, and ligaments due to their relative weakness compared to bone. Conversely, children tend to experience overuse injuries primarily in the osteochondral region [31]. The precise prevalence of OCD among football players remains uncertain. Nevertheless, there is a higher prevalence among males, which may be linked to the greater number of male children participating in football. Typically, these lesions are situated in the posterolateral aspect of the medial femoral condyle [13]. Details of studies involving baseball players are presented in Table 2. The most reported risk factors include mechanical overload (e.g., repetitive microtrauma, high training load, instability, dominant leg use, falls, quadriceps atrophy) and intrinsic factors such as young age and possible vascular insufficiency, particularly in highly active adolescents.

Prince et al. [32] in their retrospective study presented the largest group of juvenile trochlear OCD involved 34 lesions across 30 patients with an average age of 13.8 years. Within this cohort, 17 knees, accounting for 50%, were from individuals who played football. Additionally, the likelihood of patients with trochlear OCD lesions being soccer players was 2.84 times greater than that of patients with femoral OCD lesions (*p* = 0.017).

In the study by Suzue at al. [31] involving 1162 junior football players, 547 (47.1%) reported experiencing pain in the lumbar spine region or legs. Imaging examinations were conducted on 106 players (26.9%), revealing that 80 (75.5%) were diagnosed with osteochondrosis. Among them, the most prevalent form was Sever’s disease, observed in 49 players. Only one player was diagnosed with OCD, located in the femoral head.

Yanagisawa et al. [33] presented a rare case involving a 24-year-old professional soccer player who had bilateral subtalar OCD lesions on the posterior calcaneal articular surface of the talus. At the age of 16, the player experienced left lateral hindfoot pain without any apparent cause or trauma. Following imaging diagnostics one month later, lesions corresponding to stage III OCD (Berndt and Harty classification) of the talar dome were identified. After a three-month absence from practical training, the pain subsided, allowing him to return to full training without discomfort. Remarkably, there were no recurring symptoms in the left ankle for the subsequent six years. However, at the age of 20, the player developed right lateral hindfoot pain without obvious traumatic origins. Imaging revealed a lesion consistent with a Stage IV OCD lesion of the talar dome. Subsequently, he underwent operative removal of the loose bodies in his right talocalcaneal joint. Following the operation, the player rested for two weeks to facilitate wound healing. He was permitted to return to training and professional play two weeks after wound healing, with a full return to professional playing status occurring six months post-operation. The primary cause of OCD of the talar dome is typically attributed to trauma, although a vascular origin is also considered. In cases involving bilateral presentation, individual bone morphology or exercise may be ethological factors of the condition [33].

Thomas and Greig [34] presented a case involving a 17-year-old elite male soccer player who underwent surgical stabilization for an OCD lesion in the trochlea groove. The player had been experiencing progressive left-sided anterior knee pain for two months. An MRI scan revealed a cortical fracture in the trochlear groove with intact articular cartilage. The patient underwent a lateral arthrotomy with smart nailing, ensuring careful attention to avoid affecting the player’s open growth plates. Following a four-stage rehabilitation process guided by objective assessments, the player successfully returned to play 24 weeks after surgery.

Krebs et al. [35] presented a rare case of an OCD lesion in the trochlear groove of an 11-year-old female athlete, an uncommon site for OCD lesions typically found on the lateral or medial femoral condyles. The patient experienced years of intermittent knee pain that had recently worsened. Imaging with X-ray and MRI confirmed the presence of a stable lesion. Although surgical management is commonly favored in similar cases, this patient was treated successfully with a nonoperative approach. She wore a locked knee brace for 12 weeks, followed by 4 weeks of physical therapy and a 4-week gradual return to soccer activities, achieving full recovery within 5 months.

**Table 2:** OCD in football players: summary of findings.

Study	Population	Diagnostic Method	Location	Risk Factors	Treatment and Outcome
Suzue et al., 2014 [31] Prospective screening	1162 young soccer players 1 OCD case identified (0.1%)	Physical exam, ultrasound, radiographs	Distal femur	ND	ND
Deroussen et al., 2014 [36] Case report	12-year-old male	MRI	Lateral tibial condyle	Strain due to anteroposterior instability	Conservative treatment; 18-month follow-up showed osseointegration and pain-free return to sports.
D’Angelo et al., 2014 [37] Case report	13-year-old male	Clinical exam, radiographs, MRI	Medial femoral condyle	Young age Repetitive sports-related microtrauma	Multidisciplinary conservative management (education, activity modification, manual therapy, modalities, rehab) Full pain-free return to sport within ~6 months
Corominas et al., 2015 [38] Case report	14-year-old male	Clinical exam, MRI	Talar head	Repetitive stress	Failed conservative treatment; surgical retrograde drilling; casting and non-weightbearing for 6 weeks Full pain-free return to sport maintained at 5-year follow-up
Yangisawa et al., 2021 [33] Case report	24-year-old male	Radiographs, CT, MRI	Bilateral talar posterior calcaneal articular surface	Repetitive microtrauma Possible vascular cause	Left: conservative treatment successful Right: surgical excision of loose bodies Pain-free return to professional soccer at 6 months after the surgery
Cruz et al., 2021 [39] Case report	14-year-old-male	Radiographs, MRI	Patella	Repetitive microtrauma High activity level Dominant leg	Osteochondral autograft from trochlea; structured rehabilitation Pain-free with full return to play after 4 months
Krebs et al., 2021 [35] Case report	11-year-old female	Radiographs, MRI	Lateral trochlear groove	History of fall Repetitive activity	Conservative: 12 weeks in knee brace, 4 weeks physical therapy 4-week return to activity program; full pain-free return to sport at 20 weeks
Thomas et al., 2022 [34] Case report	17-year-old male	MRI, CT	Trochlear groove	Repetitive stress High training load Quadriceps atrophy	Surgical stabilization; 4-stage rehabilitation protocol; successful return to play at 24 weeks

CT—Computed Tomography; MRI—Magnetic Resonance Imaging; ND—Not Described.

### 3.3. Basketball

Basketball is considered an overhead sport, in which significant forces impact the radiocapitellar joint [40]. Typically, individuals with capitellar OCD experience a gradual increase in pain on the outer side of the elbow, a reduction in the elbow’s range of motion, and mechanical issues that could hinder athletic performance. These changes are most frequently observed in the dominant arm [40,41,42].

Basketball players may also encounter OCD in the knee. Case report by Filho et al. [16] has documented cases of bilateral OCD affecting the lateral trochlea of the femur, a condition that is less common than OCD of the medial femoral condyle. In this case, a 17-year-old amateur basketball player experienced a month of bilateral knee pain without prior trauma, worsening post-exercise. He exhibited anterior knee pain, mild edema, and crepitation, with positive Wilson’s sign. MRI revealed lesions on both femoral condyles, confirmed as stage V OCD. He underwent successful bilateral arthroscopic surgery with Herbert screw fixation. Discharged a day post-surgery, he began physiotherapy on day three, avoided weight-bearing for six weeks, and resumed sports after 16 weeks.

Benedetti et al. [43] described a 13-year-old basketball player with left knee pain, worsening after activity and improving with rest. Physical exams indicated plica synovialis, but MRI revealed juvenile OCD of the lateral femoral condyle. Given a 29% risk of bilateral OCD, the asymptomatic right knee was also imaged, confirming a similar lesion. The patient underwent 6 months of physical therapy and reduced sports intensity, leading to pain resolution and no lesion progression on follow-up MRI. This case highlights the need to assess for bilateral OCD in unilateral cases and supports early conservative management.

### 3.4. Gymnastics

The occurrence of OCD in gymnasts is well-described in the literature [15,44,45,46,47]. The elbow is the most commonly affected site, primarily at the capitellum, followed by the radial head [48]. In the systematic review by Westermann et al. [49], which included 492 athletes treated for capitellar OCD, 7.5% were gymnasts. Gymnasts usually bear weight in a position with their arms fully extended overhead, resulting in force directed almost straight down. Therefore, OCD lesions in gymnasts typically appear about 30° further back on the capitellum than in baseball players [40,50]. OCD of the capitellum predominantly female gymnasts which typically develop symptoms at a younger age than males (13.6 compared to 15.1 years) and show symptoms in both elbows equally. In contrast, 80% of male gymnasts exhibit OCD lesions in their dominant elbow [51]. Upon examination, there may be slight loss of elbow extension accompanied by tenderness on the outer elbow, possibly with crepitus during movement, particularly noted during pronation and supination. Pain reproduced at the radiocapitellar joint during active pronation and supination with an extended elbow indicates a positive radiocapitellar compression test. There may also be swelling of the elbow joint [15,41,52]. Details of studies involving baseball players are presented in Table 3.

In the cohort study by Clark [24], gymnasts presenting with capitellar osteochondritis dissecans (COCD) demonstrated unique lesion characteristics likely related to the impact of weightbearing activities on the elbow. Among the 126 elbows reviewed, gymnasts accounted for 68 cases, with 70% of patients with bilateral COCD being gymnasts, indicating a significant correlation between gymnastics participation and bilateral lesion formation (*p* = 0.036). Lesions in gymnasts were more posteriorly positioned (mean sagittal inclination angle: 41.2° ± 14.9°) and exhibited less lateral containment (3.6 ± 1.9 mm) compared to non-upper extremity athletes (*p* = 0.001 and *p* = 0.015, respectively). These findings underscore the unique biomechanical demands of gymnastics and their potential role in lesion development and positioning.

In the cohort study by Zheng et al. [53], 154 patients with capitellar osteochondritis dissecans were prospectively evaluated to assess the outcomes of surgical treatment based on a framework considering lesion containment and subchondral bone disease depth. Patients, primarily athletes (39% gymnasts, 28.5% baseball/softball players), underwent drilling/microfracture, internal fixation, or autologous osteochondral grafting (OG). Clinically and functionally, significant improvements in pain, elbow motion, and Timmerman scores were observed postoperatively, with 76% returning to their primary sport. OG had lower revision rates and superior functional outcomes, particularly in Nelson grade 2 lesions, compared to other techniques, highlighting its potential even for lower-grade OCD lesions.

Yehyawi et al. [54] studied retrospectively 55 young competitive gymnasts, treating 69 elbows for OCD lesions surgically. The average age at surgery was 12.1 years, and 33% were at a pre-elite gymnastics level. A total of 29% underwent surgery on both elbows. The average lesion size was 10 mm. Treatments involved debridement and microfracture in 78% of cases, and debridement alone in 22%. In total, 90% returned to competitive gymnastics at the same or higher level, although 97% experienced some difficulties with specific events post-recovery.

### 3.5. Clinical Applications

The management of OCD requires a collaborative approach involving a multidisciplinary team that includes a radiologist, orthopedic surgeon, physical therapist, nurse practitioner, and primary caregiver. Early and accurate diagnosis coupled with appropriate management strategies is essential for effective treatment outcomes [10]. The clinical manifestations of OCD are varied and depend largely on the stage and severity of the lesion as well as the specific joint involved (Table 4). Stable lesions often result in nonspecific symptoms such as swelling, pain related to activity or upon palpation, a mild grinding sensation, limited range of motion, and joint effusion. On the other hand, unstable lesions or loose bodies can cause symptoms such as the joint catching, clicking, or locking [10].

Treatment strategies vary based on several factors including the patient’s age, the timing of the diagnosis, the severity of symptoms, and the stability of the lesion. For stable lesions in younger patients, conservative treatments are frequently advised, including immobilization and protected weight-bearing. These non-surgical approaches involve using casts, braces, or splints to immobilize the affected area, limiting weight-bearing activities, and engaging in muscle-strengthening exercises. Physiotherapy techniques, such as extracorporeal shock wave therapy are also incorporated. Typically, these conservative treatments are applied over a duration of three to six months and varies depending on which joint is affected [10,61,64,65].

When conservative treatments have not desired results in stable lesions, surgical options may be considered. Drilling techniques, either retroarticular or transarticular, are employed, with transarticular drilling often achieving higher success and symptom relief rates. Unstable or displaced lesions typically necessitate surgical intervention, commonly via arthroscopic methods. Outcomes for stable lesions are generally more favorable than those for unstable lesions [10,66,67]. Table 5 compiles the most common surgical treatment methods for patients with knee, elbow, and ankle OCD, the most frequent locations of the condition.

While both treatment options involve physical therapy, the approach and intensity can vary. Non-surgical therapy emphasizes joint protection and gradual healing through conservative measures, whereas post-surgical therapy is more focused on recovering from the surgical intervention and restoring full joint function. Post-surgical rehabilitation may require a more intensive and prolonged therapy regimen to address surgical recovery and ensure proper healing [68,69].

## 4. Discussion

In our review we gained valuable insights into the varying risks and prevalence of OCD among different athletic disciplines. By reviewing the literature, we identified specific sports where athletes are more prone to developing OCD, highlighting the unique biomechanical demands and repetitive stress factors contributing to these risks. This comprehensive understanding allows for better prevention strategies, tailored interventions, and informed decision-making by healthcare providers, coaches, and athletes. Additionally, our findings underscore the need for sport-specific research and targeted clinical practices to mitigate the incidence of OCD in vulnerable athletic populations.

The occurrence of OCD varies significantly across different sports, with the highest prevalence observed in baseball and gymnastics. It should be noted that cultural differences in sports participation across regions may influence the reported prevalence of OCD, as certain sports are more popular and, thus, more studied in specific populations. This variation underscores the importance of understanding the sports-specific risks and the necessity for targeted prevention and management strategies. Moreover, the location of OCD is closely associated with the joints most frequently used in a particular sport, highlighting the need for sport-specific conditioning and protective measures. The risk factors identified across the included studies were largely consistent and can be grouped into extrinsic and intrinsic categories. Extrinsic factors primarily involved repetitive mechanical stress related to sport-specific movements, such as high training load, dominant leg use, repetitive jumping or kicking, and prior trauma or falls. These were especially noted in sports like soccer, gymnastics, and baseball. Intrinsic factors included young age, skeletal immaturity, and in some cases, suspected vascular insufficiency. The convergence of high mechanical demands and biological vulnerability in young athletes likely underlies the development of OCD lesions across various anatomical sites.

Prevention of OCD in young athletes remains a key challenge in sports medicine, especially in disciplines with high joint-loading demands such as baseball, gymnastics, and football. Other sports also likely carry a risk of OCD; however, there is too little data available in the literature to draw meaningful conclusions. Current evidence suggests that several modifiable risk factors can be targeted to reduce the incidence and progression of OCD. One of the most consistent recommendations is to delay early sports specialization, particularly in overhead or high-impact sports. Specializing in a single sport before the age of 12 has been associated with an increased risk of overuse injuries, including OCD, due to repetitive loading of vulnerable joint surfaces during skeletal immaturity [70,71,72]. Training load management is also critical. Structured guidelines recommend limiting repetitive movements (e.g., pitch counts), incorporating at least one to two rest days per week, and ensuring athletes take at least three months off per year from their primary sport to allow for recovery and skeletal adaptation [71,73]. Additionally, proper instruction in sport-specific biomechanics such as throwing mechanics in baseball or landing techniques in gymnastics can help minimize joint shear and compressive forces. Targeted neuromuscular training and strength programs that improve core and limb control may further reduce the risk of overload [74]. Clinicians and coaches should also be educated to recognize early signs of overuse injury, including extension pain, joint effusion, or limited range of motion, and should not encourage athletes to play through pain. Routine clinical screening in high-risk groups and, in some cases, imaging may aid in early detection and prevent progression to unstable lesions. Importantly, training intensity should be aligned with the athlete’s biological age and skeletal development, not just chronological age, as periods of rapid growth are associated with greater vulnerability of the subchondral bone [70,75].

The prognosis for athletes, particularly adolescents, recovering from OCD is generally positive, with a high rate of return to sport. This finding offers encouragement to affected athletes, reinforcing that OCD lesions, while challenging, do not necessarily signify the end of a sporting career. However, it is crucial to set realistic expectations for recovery. Athletes may not experience a fully asymptomatic return to all events within their sport, indicating the necessity for a carefully planned, stepwise approach to rehabilitation and return to play.

The duration of symptoms before treatment has been found to correlate with the severity of OCD, underscoring the importance of early detection and intervention. Consequently, primary care clinicians, particularly physical therapists, play a pivotal role in the early identification of suspected pathological conditions. Physical therapists, especially those working in direct access settings, must be vigilant for subtle signs and symptoms of OCD and other significant pathologies. Awareness of specific risk factors and the ability to recognize the early stages of these conditions can facilitate timely referral for imaging, medical assessment, or surgical intervention, thus preventing progression and facilitating a more favorable outcome for the athlete.

In sum, understanding the sport-specific risks of OCD, ensuring early intervention, and adopting a cautious, stepwise return to sport are critical for managing this condition effectively. By fostering a collaborative approach among athletes, coaches, and healthcare professionals, we can optimize recovery, ensure safe return to play, and ultimately, safeguard the health and careers of athletes. However, despite increasing awareness of the risk factors and mechanisms behind OCD, high-quality prospective studies are still needed to establish and validate specific prevention strategies. Future research should focus on identifying effective interventions such as training modifications, load monitoring protocols, and biomechanical correction programs that can reduce the incidence of OCD across various sports and age groups.

We focused primarily on baseball, basketball, gymnastics, and football, as these sports are the most frequently represented in the available literature on OCD. While other sports may also pose a risk, the number of published reports is currently too limited to allow for meaningful analysis. This represents a limitation of our review, as the findings may not fully reflect the broader spectrum of sports-related OCD. Other limitation of our review is heterogeneity of included studies because case studies, original articles, and review articles were mixed. Furthermore, age- and sex-related differences, training stages, and body composition of the athletes were not analyzed, which causes some difficulties in interpreting the results of this study. In addition, we used only two databases—PubMed and Embase—to which we had institutional access. However, these two sources cover a substantial portion of the relevant medical literature.

## 5. Conclusions

OCD is a rare but clinically significant condition affecting young athletes, with sport-specific patterns of lesion location and risk factors. This narrative review highlights the importance of early recognition, the role of repetitive mechanical stress in pathogenesis, and the variability in treatment approaches. Although high-quality evidence remains limited, especially in less common sports and anatomical sites, current findings support sport-specific monitoring and prevention strategies. Further research, including prospective and comparative studies, is needed to establish evidence-based diagnostic and treatment pathways.

## Figures and Tables

**Table 3:** OCD in gymnasts: summary of findings.

Study	Population	Diagnostic Method	Location	Risk Factors	Treatment and Outcome
Clark et al., 2024 [24] Cross-sectional study	A total of 126 elbows were identified: 68 in gymnasts Mean age of the participants was 12.5 years	Radiographs, MRI	Humeral capitellum	ND	ND
Zheng et al., 2024 [53] Cohort study	154 patients were prospectively enrolled—39% were gymnasts Mean age of the participants was 13.7 years	Clinical exam, MRI	Humeral capitellum	ND	145 elbows treated surgically (drilling, fixation, OG grafts); improved pain, motion, symptoms 76% returned to primary sport
Yehyawi et al., 2024 [54]	55 adolescents (in a total of 69 elbows) Average age at time of surgery was 12.1 years	Clinical and radiologic	Humeral capitellum	ND	40 elbows: 78% debridement + microfracture, 22% debridement alone 90% returned to competitive gymnastics at same or higher level

**Table 4:** Symptoms and treatment approaches for osteochondritis dissecans by stage [55,56,57,58,59,60,61,62,63].

Site	Stage	Symptoms	Treatment
Knee	Early	non-specific pain during physical activity	reducing physical activities and limiting participation in sports immobilization weight-bearing activities
	Advanced	progressive increase in stiffness swelling catching or locking loss of range of motion is not typically observed	subchondral drilling internal fixation microfracturing autologous chondrocyte implantation bone marrow stimulation bone marrow-derived cells transplantation
Elbow	Early	mild pain tenderness swelling on the outer side of the elbow	same as in the knee
	Advanced	loss of extension in the elbow intermittent episodes of catching and locking	fragment fixation cancellous bone grafts pull-out wire technique osteochondral autograft debridement bone marrow–derived cells transplantation hyaluronic acid-based scaffold
Ankle	Early	intermittent pain during weight-bearing activities	same as in the knee
	Advanced	intense pain swelling in the joint instability during walking joint locking	subchondral drilling microfracturing chondrocyte implantation
Shoulder	All	pain motion limitation	conservative treatment in early stages drilling of the sclerotic margin intra-articular debridement
Hip	All	pain catching and locking	conservative treatment in early stages surgical removal of the free OCD fragment internal fixation osteochondral autograft transplantation

**Table 5:** Examples of treatment methods for various locations of OCD.

Knee OCD	Elbow OCD	Ankle OCD
subchondral drilling internal fixation using screws, anchors, arrows, and pins microfracturing autologous chondrocyte implantation bone marrow stimulation bone marrow-derived cells transplantation	fragment fixation using Herbert’s screws, or bioabsorbable screws cancellous bone grafts pull-out wire technique osteochondral autograft transfer debridement bone marrow–derived cells transplantation hyaluronic acid-based scaffold	subchondral drilling microfracturing chondrocyte implantation techniques

## Data Availability

Data sharing is not applicable. No new data were created or analyzed in this study.
